# Molecular Cloning, Characterization, and Expression Analysis of an Ecdysone Receptor Homolog in *Teleogryllus emma* (Orthoptera: Gryllidae)

**DOI:** 10.1093/jisesa/iev010

**Published:** 2015-03-22

**Authors:** Hui He, Gengsi Xi, Xiao Lu

**Affiliations:** Institute of Zoology, College of Life Sciences, Shaanxi Normal University, Xi'an, 710062, People's Republic of China

**Keywords:** molecular clong, ecdysone receptor (EcR), *Teleogryllus emma*, real time RT-PCR

## Abstract

Ecdysteroids are steroid hormones that play important roles in the regulation of Arthropoda animal growth development, larvae ecdysis, and reproduction. The effect of ecdysteroids is mediated by ecdysteroid receptor (EcR). The ecdysone receptor (EcR) belongs to the superfamily of nuclear receptors (NRs) that are ligand-dependent transcription factors. Ecdysone receptor is present only in invertebrates and plays a critical role in regulating the expression of a series of genes during development and reproduction. Here, we isolated and characterized cDNA of the cricket *Teleopgryllus emma* (Ohmachi & Matsuura) (Orthoptera: Gryllidae) and studied mRNA expression pattern using real time-polymerase chain reaction. The full-length cDNA of *T. emma* EcR, termed TeEcR, is 2,558 bp and contains a 5′-untranslated region of 555 bp and a 3′-untranslated region of 407 bp. The open reading frame of TeEcR encodes deduced 531-amino acid peptides with a predicted molecular mass of 60.7 kDa. The amino acid sequence of *T. emma* EcR was similar to that of known EcR especially in the ligand-binding domain of insect EcR. Real-time quantitative reverse transcription-polymerase chain reaction was performed to compare TeEcR mRNA expression level at the whole body and gonad during *T. emma* development. The data revealed that TeEcR mRNA is differentially expressed during *T. emma* development, with the highest expression level in late-instar larvae of the body and lowest in third instar. The levels of TeEcR transcripts also vary among gonads development, and levels in ovaries were higher than in testes at every developmental stage. These results suggest that TeEcR may have potential significance to regulate the morphological structure and gonad development of *T. emma*, due to its expression in different developmental periods.

Ecdysteroids are invertebrate-specific steroid hormones secreted only by Arthropods that trigger a wide variety of developmental and physiological processes such as shedding, molting, metamorphosis, and reproduction in *Drosophila melanogaster Meigen *, *Bombyx mori (L.),* and other arthropods (Riddiford 1993, Subramoniam 2000, Truman and Riddiford 2002, Sekimoto et al*.* 2006).

The ecdysteroid signals are transduced to target genes via heterodimeric complex of the ecdysteroids receptor (EcRs) and the retinoid X receptor homolog ultraspiracle (USP), both members of the nuclear receptor (NR) superfamily (Yao et al. 1993). The EcR/USP complex binds to the responsive element with a specific nucleotide sequence to elicit the expression of early ecdysone responsive genes, such as *E74* and *E75*, and eventually to regulate further downstream transcriptional cascades (reviewed by King-Jones and Thummel [2005]). Recently, it has been also reported that EcR can regulate the transcription of the target genes without USP, this may indicate the action of EcR homodimer or heterodimer of EcR with a novel partner (Grebe et al. 2004, Ogura et al. 2005, Costantino et al. 2008).To date, a number of complete *EcR* cDNA sequences have been cloned and characterized primarily in insects, such as Diptera [*Lucilia cuprina* (Wied.) (Hannan and Hill 1997)]; Coleoptera [*Tribolium castaneum* Herbst (Tan and Palli 2008), *Leptinotarsa decemlineata* (Say) (Ogura et al. 2005)]; and Lepidoptera [*Choristoneura fumiferana* (Clem.) (Kothapalli et al. 1995), *Spodoptera litura* (F.) (Nagata et al. 2005)]. The expressions of the *EcR* gene and its potential roles during molting also have been extensively investigated. In *Drosophila*, three different EcR isoforms have been identified and shown to predominate in different target tissues, and at different developmental stages (Talbot et al. 1993). The appearance of two different forms in the moth, *Manduca sexta* (L.), is also modulated during development. In both dipterans and lepidopterans, ecdysone can act through the EcR isoforms in combination with another receptor superfamily member, the USP protein (Kothapalli et al. 1995). As yet, however, few primitive insect NR sequences have been described except these more advanced insect orders.

We have now identified an NR from the more primitive, exopterygote insect, *T*. *emma,* named TeEcR, that encodes the ecdysone receptor of this species. TeEcR mRNA expression patterns at the embryo, distinct developmental stages of the whole bodies and gonads were studied by real-time quantitative reverse transcription-polymerase chain reaction (RT-PCR). Sequence and expression studies show a clear relationship with previously described ecdysone receptors.

## Materials and Methods

### 

#### Insects

The Chinese field crickets *T. emma* were obtained from a commercial supplier. This species typically undergoes six instar stages before maturity. The crickets were raised at 26 ± 1°C temperatures, a relative humidity of 60% and a photoperiod of 12:12 (L:D) h. The crickets were fed with goldfish flakes and fresh lettuce every 2 d. Water was provided via Petri dishes filled with wet cotton wool, which was also replaced every 2 d. Embryos, larvae from one to six instars, adults of male and females, testes and ovaries from fourth-instar larvae to adults were collected, and five individual were used, respectively. They were immediately frozen in liquid nitrogen and stored at −80°C preparing for RNA extraction.

#### Molecular Cloning of TeEcR

Total RNA was extracted using Takara RNAiso Plus reagent (Invitrogen, Takara Bio Inc., Shiga, Japan) according to the manufacturer's protocol and then immediately reverse transcribed for the generation of cDNA using a First Strand cDNA Synthesis Kit with oligo(dT) primer (Fermentas Life Science, Burlington, Ontario, Canada; http//www.frementas.com/) following the manufacturer’s instruction.

The full-length cDNA of TeEcR was cloned according to the scheme shown in Table 1. A cDNA fragment of TeEcR was amplified using degenerate primers A1 and A2 for first PCR; B1 and B2 for nested PCR. The design of which was based on the conserved sequence of DNA-binding domain (DBD) and ligand-binding domain (LBD) of insect EcR (Table 2)*.* Inosine was used to reduce primer mismatch. PCR amplification was performed in 50 μl reaction volumes with the following protocol: 95°C for 3 min, followed by 32 cycles of 95°C for 50 s, different melting temperatures for 45 s, 72°C for 30 s, and a final extension at 72°C for 10 min. PCR products of the expected size were purified from agarose gel using a Gel Extraction Kit (Axygen Scientific Inc., San Francisco, CA., USA; http//www.axygen.com/) and subcloned into the pMD19-T simple vec tor using a TA Cloning kit (Takara Bio Inc., Shiga. Japan; http//www.takara-bio.com/). Three PCR-positive colonies were selected for sequencing.

**Table 1.** Oligonucleotide primers used for cDNA cloning and real-time quantitative reverse transcription polymerase chain reaction

Target	Name	Primer sequence 5’–3’	Expected size (bp)	Annealing temp. (°C)
EcR	A1	AGCTAYGAYCCSTACAGYCC	224	52
	A2	CTBTGYCTBGTNTGYGGVGAC		
	B1	ATRAGYTCYTCYTGYTCHGG		
	B2	CACAYTCYGGCCTCATSCCVAC		
	C1	GTBGGSATGAGGCCRGARTGTG	643	50
	C2	CCDGARCARGARGARCTYAT		
	D1	TCCCAKATYTCVGCVAGGAA		
	D2	GAGAARCACATYTCBGARTT		
EcR 3’-RACE	Oligo	GCTGTCAACGATACGCTACGTAACGGCATGACAGTG(T)18	432	53
	Outer	GCTGTCAACGATACGCTACGTAACG		
	GSP1	CAGAACTCVRAVATGTGCWTC		
	Inner	CGCTACGTAACGGCATGACAGTG		
	GSP2	TTCCTBGMBGAGATCTGGGA		
EcR 5’-RACE	outer	CATGGCTACATGCTGACAGCCTA	1,076	55
	GSP1	ATTCTTTGTAATGCTCCTCC		
	Inner	CGCGGATCCACAGCCTACTGATGATCAGTCGATG		
	GSP2	TCGCAGGTGAGTGCATTGTAG		
EcR real-time	F	TGCTTACAGAATTAAGGACACTTGG	91	60
	R	GCGAGGAAAGGAGGCAGTT		
β-actin	F	CCCTCTTCCAGCCATCGTTC	250	60
	R	CCACCGATCCAGACGGAGTA

**Table 2.** Possessing EcR genes’s insect species name and abbreviation used to construct phylogenetic in this study

Classification	Species	GenBank accession no.	Product size (a.a)	Identity (%)
Hymenoptera	*Apis mellifera* L. (Honey bee), Ame	NP-001091685	629	74
	*Camponotus japonicus* Mayr (Cja)	BAF79666	504	78
	*Pheidolemegacephala* F. (Pme)	BAE47509	633	70
	*Nasonia vitripennis* (Walker) (Nvi)	NP-001152828	577	77
Coleoptra	*Tribolium castaneum* Herbst (Tca)	NP-001107650	549	89
	*Leptinotarsa decemlineata* (Say) (Lde)	BAD99296	565	80
	*Tenebrio molitor* L. (Tem)	CAA72296	491	86
	*Anthonomus grandis* Boheman (Agr)	ACK57879	479	80
Diptera	*Drosophila melanogaster* Meigen (Dme)	NP-724456	849	65
	*Calliphora vicina* R.-D. (Cav)	AF325360	784	62
	*Ceratitis capitata* (Wiedemann) (Cca)	CAA11907	673	66
Lepidoptera	*Bombyx mori* L. (Bmo)	NP-001037331	543	61
	*Plodia interpunctella* (Hubner) (Pin)	AAR84611	541	64
	*Spodoptera litura* F. (Sli)	ABX79143	588	65
	*Choristoneura fumiferana* (Clem) (Cfu)	AAC61596	513	63
Orthopteroidea	*Locusta migratoria manilensis* Meyen (Lmi)	AAD19828	541	92
	*Blattella germanica* L. (Bge)	CAJ01677	570	87
Phthiraptera	*Pediculus humanus corporis* De Geer (Pco),	EEB17490	520	87
Hemiptera	*Nilaparvata lugens* Stal (Nlu)	ACO55652	688	72

The fragments obtained were compared with the published insect sequences to confirm the homology with others. Based on these initial sequences, further specific and degenerate primers (C1 C2 D1 D2) (Table 1) were designed and used to obtain the other TeEcR gene coding sequence.

The full-length cDNA of TeEcR was amplified by 3′- and 5′-rapid amplification of cDNA ends (RACEs) using 3′-Full RACE Core Set and 5′-Full RACE Core Set (Invitrogen, Takara Bio Inc., Shiga, Japan), following the manufacturer’s instruction. The cDNA was amplified with the gene-specific primer (GSP1) (Table 1) and 3′-RACE outer primer. Nested PCR was performed with the gene-specific primer (GSP2) (Table 1) and 3′-RACE inner primer. 5′-RACE is the same as 3′-RACE used the two pairs of gene-specific primers (GSP3, 4) (Table 1). PCR reactions were performed as described above except for different templates and annealing temperatures (Table 1).

#### Structure and Phylogenetic Analysis of TeEcR

The open reading frame (ORF) of TeEcR was obtained by means of the online tool National Center for Biotechnology Information ORF Finder (http//www.ncbi.nlm.nih.gov/gorf/gorf.html). Signal peptide prediction was performed using the SignalP program (Centre for Biological Sequence Analysis; http//www.cbs.dtu.dk./services/SignalP/; Bendtsen et al. 2004). Potential functional motifs of the protein sequence were analyzed using the PROSITE database (Expert Protein Analysis System. Swiss Institute of Bioinformatics, Basel; http//myhits.isb-sib.ch/cgi-bin/motif_-_scan). The deduced amino acid sequences of TeEcR were aligned with the known EcRs using the ClustalW program. Phylogenetic analyses were conducted using MEGA version 4.0 (Tamura et al. 2007). The phylogenetic trees were constructed by the neighbor-joining method using LBDs of EcRs.

#### Real-Time Quantitative RT-PCR

To determine *EcR* transcript expression patterns, the quantitative real-time RT-PCR was performed in different development stages and different gender, including eggs a month developing, larvae from one to six instar 3 d developing, male and female adults 10th d developing, and the gonad was dissected out from each cricket. Testes and ovaries from fourth-instar larvae to adults were collected 3 d developing. Samples were taken from five individuals, respectively. Each reaction included 1 µl of cDNA and 0.2 µM primer (Te-EcR-real-F/R and β-actin-real-F/R (Table 1). Reactions were performed using iQ5 apparatus (Bio-Rad Laboratories, Inc., Hercules, CA; http://www.bio-rad.com) with a SYBR Premix Ex Taq Kit (Takara Bio Inc.) and the detailed protocol was as follows: 95°C for 1 min, 40 cycles of 95°C for 10 s, and 59°C for 25 s, followed by a dissociation-curve program from 55 to 95°C with a heating rate of 0.5°C every step and continuous-fluorescence acquisition.

One of the cDNA samples was used to construct standard curves for TeEcR and β-actin after serial dilution and the slopes of the curves were obtained. Expression levels were determined using the formula *F* = $10^{\Delta C_{\text{t,t}}/A_{\text{t}}-\Delta C_{\text{t,r}}/A_{\text{r}}}$proposed by Zhang et al*.* (2005), where *F* is the relative expression of the samples, *C*_t_ is the number of cycles necessary to reach a defined fluorescence threshold, *A*_t_ and *A*_r_ are the slopes of the TeEcR and β-actin standard curves, respectively, and △*C*_t.t_ and △*C*_t.r_ denote the difference between the *C_t_* value of samples and the calibrator for TeEcR and β-actin, respectively, and we designated the *F* value of the calibrator as 1. The normalized amount (TeERR/β-actin) is deduced from the C_t.t_ and C_t.r_ of the calibrator sample to obtain the difference between △*C*_t.t_/*A*_t_ and △*C*_t.r_/*A*_r_. Here, the cDNA of embryos was selected as the calibrator in analyzing TeERR expression at different development stages of the whole body. For analyzing TeERR expression in the gonads, the gonads of fourth-instar male were selected as the calibrator. Measurements were performed in triplicate using the pooled samples. Date was entered into an Excel spreadsheet (Microsoft Corp.: http://www.microsoft.com) to obtain *F* values. Analysis of the data was carried out using SPSS 13.1 (SPSS Inc. 2004).

## Results

### 

#### Cloning and Characterization of TeEcR cDNA

**Fig. 1.**
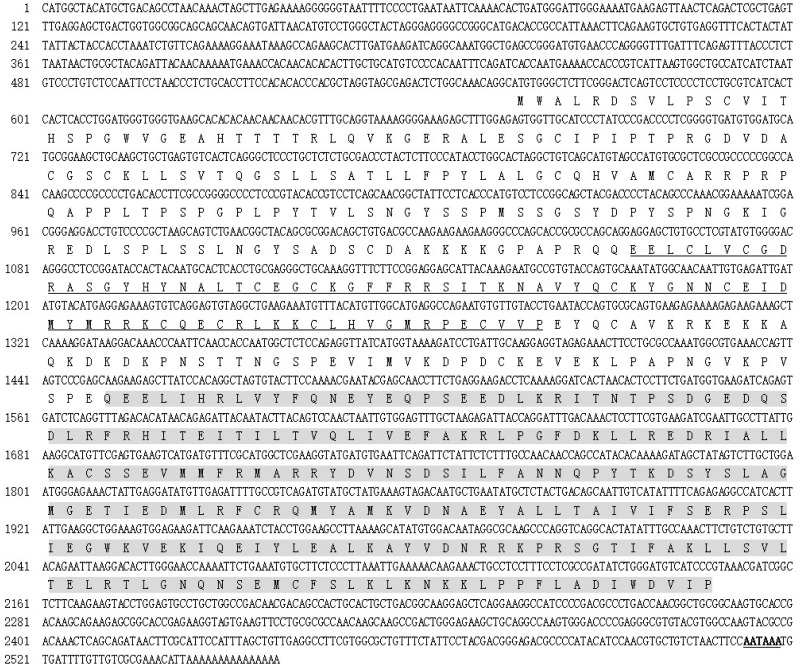
Complete cDNA sequence and deduced amino acid sequence of the cricket *T. emma* EcR gene. The modified poly(A) signal sequence(AATAAA) is underlined. The conserved DBD (domain C, 164–236 a.a.) is underlined and the LBD (domain E, 321–545 a.a.) are indicated with shadow.

**Fig 2.**
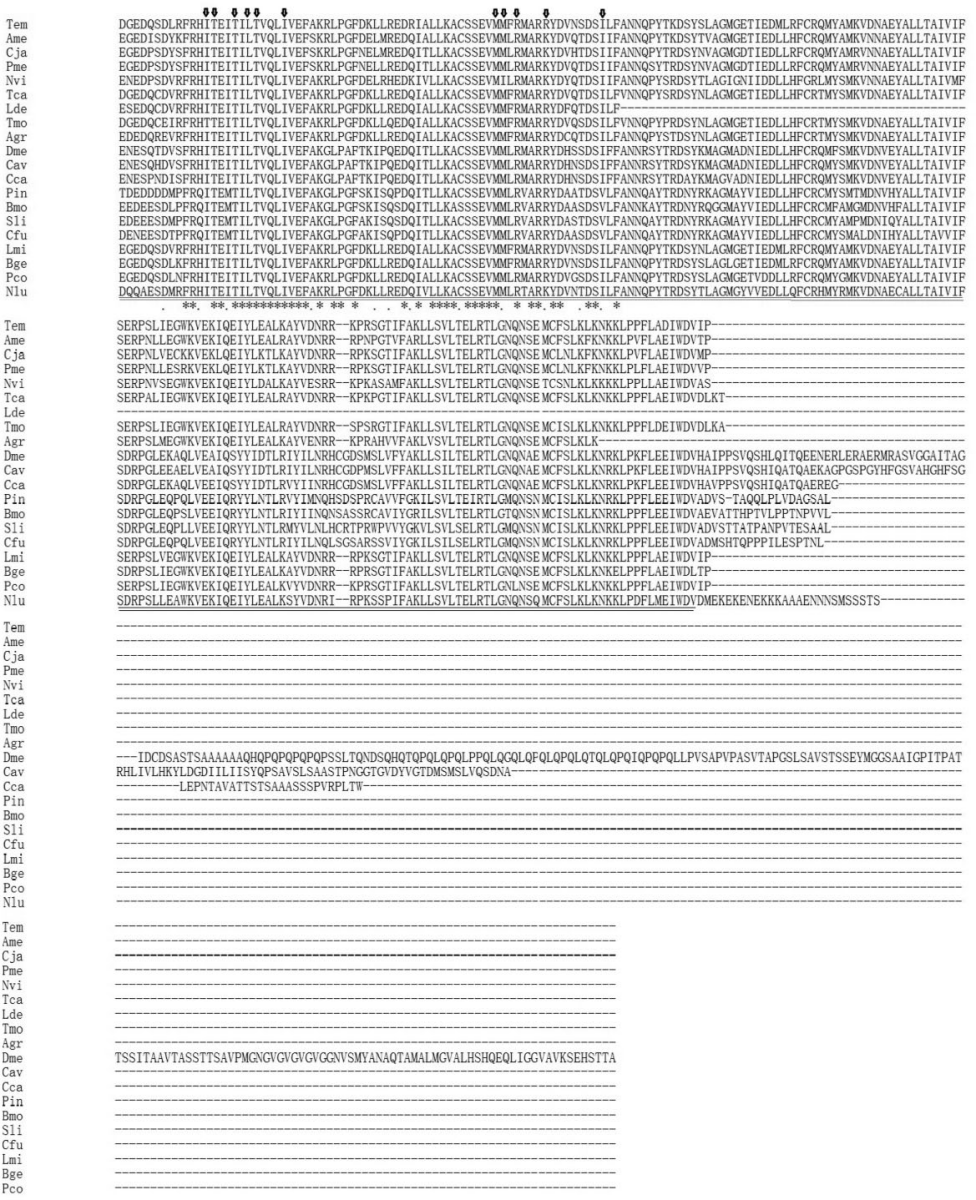
Alignment of the predicted amino acid sequence of TeEcR with the orthologs from various organisms (Table 2). Conserved amino acids in all EcR are shown in black and residues that are similar with respect to side chains in gray; gaps are introduced to optimize the alignment. The DBD is underlined and the LBD is double underlined. The cysteine residues of the zinc finger motifs in the DBD are indicated by asterisks. The P-box (E188 to G192) and the D-box residues (K207 to N211) that are important for the binding to hormone response element are underlined in ref. The arrow heads represent the conserved amino acid residues among insects for the interaction with EcR and ponasteron A.

A 224-bp fragment was amplified first by RT-PCR using degenerate primers (Table 1) designed from the highly conserved regions of the DBDs and LBDs of several insect EcRs, and the nucleotide sequence was converted to an amino acid sequence. The deduced amino acid sequence from the PCR product was similar to the corresponding EcR region of insects. Based on this fragment, special upstream primers C1 C2 was designed to obtain another fragment about 643 bp using nest PCR. Subsequently, the full length of the cDNA sequence was determined by 5′-RACE and 3′-RACE. Basic Local Alignment Search Tool (BLAST) searches indicated that the deduced amino acid sequences were analogous to the cockroach *Blattella germanica* EcR (83% identity) and locust *Locusta migratoria* EcR (79%). Therefore, this sequence was confirmed as the *Te*EcR and deposited in GenBank (accession no. GQ351503). The *TeEcR* cDNA is 2,558 bp in length, including 555 bp of 5′-untranslated region (5′-UTR) and 407 bp of 3′-UTR with the modified poly (A) signal sequence AATAAA (Fig. 1). The ORF encodes 531 amino acids (a.a.) with a molecular weight of 60.7 kDa. The amino acid sequence alignment indicated that this *Te*EcR includes the five domains (A/B, C, D, E, and F). The amino acid sequence alignment indicated that this EcR polypeptide included the entire A/B (1–166), C (164–236), D (243–298), and E/F (321–545) regions (numbers in parentheses indicate the first and last amino acids of the primary sequence of the proteins). These five domains normally present in steroid receptor superfamily members (Krust et al. 1986, Evans 1988). Especially, the C-region (DBD) and E/F-region (LBD) including critical amino acid residues for recognition of DNA-binding sites and interaction with ligands are highly conserved with those of other insects (Fig. 2).

#### Alignment Analysis and Phylogenetic-Tree Construction

We compared the deduced amino acid sequences of TeEcR with those of EcRs from other insects (Table 2). TeEcR is most similar to the EcR of locusta *L.* *migratoria* (92% identity), followed by *B.* *germanica*, *T.* *castaneum*, and *P.* *corporis* (89–87% identity). We also compared A/B, C, D, and E regions of EcRs among insects (Fig. 2). It showed that the C region of EcRs is highly conserved among the insects (89–100%), but the amino acid sequences of E regions varied among the insects. The sequence of the E region of TeEcR is highly analogous to that of *L. migratoria, B.* *germanica* EcR (93% identity) and *T.* *castaneum*, *P.* *corporis* EcR (90% identity), and moderately analogous to those of Hymenoptera and Coleoptera (87–70%). The identity of the A/B regions of EcRs is not as high as the identity of the C and E regions (<41%). The high conservation of the residues involved in ligand binding suggests that TeEcR may have an ability to bind ecdysteroids.

**Fig 3.**
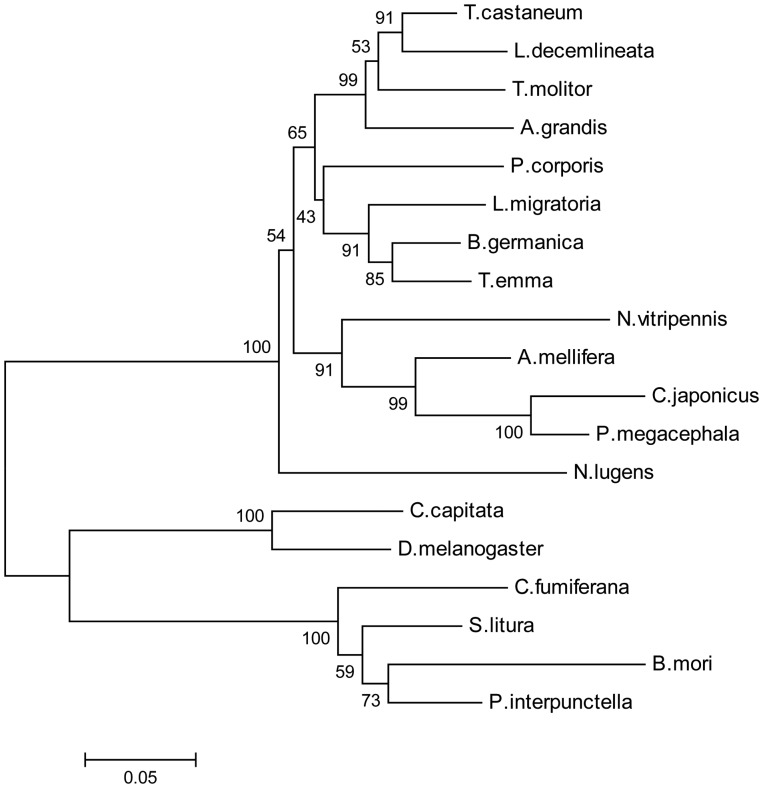
Phylogenetic tree constructed on the basis of alignment of the amino acid sequences of EcR homologs and showing the evolution relationship of TeEcR with other insects of the EcR family. Numbers at branch nodes are percentages of bootstrap confidence values derived from 2,000 replications.

The phylogenetic trees including known homologs of EcR protein sequences (Table 2) were constructed using the neighbor-joining method with Poisson correction (Fig. 3). Results from phylogenetic revealed that TeEcR shared about 92% identity with *Locusta migratoria* EcR; followed by 80–89% identity with Coleoptera EcR*;* then 70–78% identity with Hymenoptera EcR; and 61–66% identity with Diptera and Lepidoptera EcR*.*

#### Analysis of TeEcR mRNA Expression

**Fig 4.**
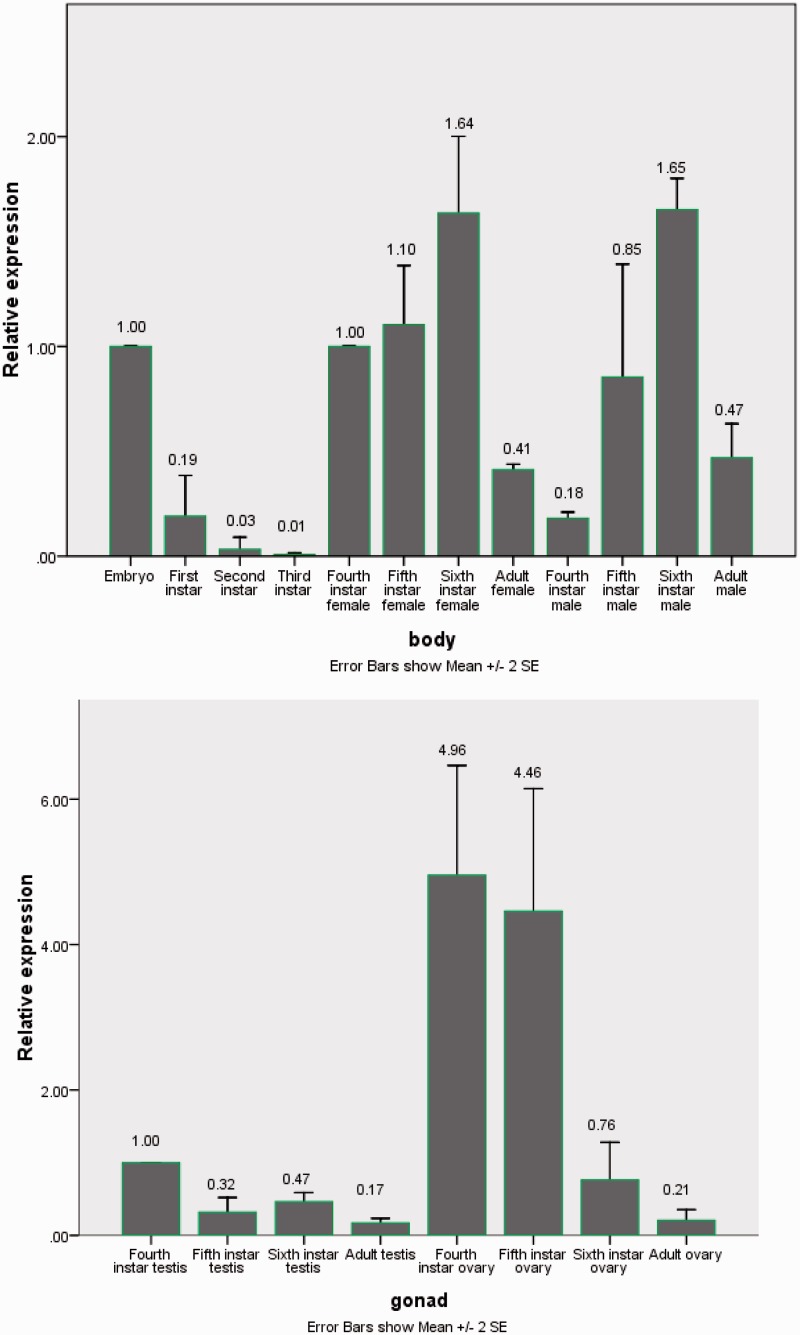
Relative expression profiles (mean ± 2 SEM, *n* = 3) of the TeEcR gene, determined by real-time PCR. (A) TeERR mRNA at the whole body during different development stages. All expression levels are shown relative to the expression level in the embryos. (B) TeEcR mRNA at gonad during different development stages. All expression levels are shown relative to the expression level in fourth-instar male.

Total RNA extracted from embryos, whole body of one to six larvae, males, females, testes, and ovaries was performed by real-time quantitative RT-PCR. Changes in the expression levels of *TeEcR* were investigated at different development stages. Previous studies clearly indicated that the analyzed β-actin mRNA levels remain fairly constant in tissues of insects regardless of their developmental or physiological condition (Claeys et al. 2003, Simonet et al. 2004). Consequently, we selected the *T. emma* β-actin (AB055975) gene as an internal standard of housekeeping transcript in real-time PCR analysis. To obtain precise quantification, the specific PCR products and the absence of primer dimers were confirmed by viewing the single peak in the melting curve if the genes (*TeEcR* and β-actin) tested (unpublished data). TeEcR relative expression levels were calculated using the formula *F* = $10^{\Delta C_{\text{t,t}}\text{/}A_{\text{t}}-\Delta C_{\text{t,r}}/A_{\text{r}}}$ for each replicate The results displayed that TeEcR was expressed in all samples at different levels both the bodies and gonads (Fig. 4). In the whole body of different development stages, TeEcR gene was found to be expressed gradually decreased from embryo to third instar and then significantly increased from fourth instar to sixth instar, but the expression was decreased again in the adult. Compared with male, the expression of TeEcR gene in female was higher during fourth and fifth-instar larvae development stage but lower in sixth-instar larvae and adults.

The expression of TeEcR in the testes was higher in fourth-instar larvae, then decrease in fifth instar, but increase a little in sixth-instar larvae and down to the bottom in the adult, which expression pattern was different from the ovaries (Fig. 4). In the ovaries, the expression of TeEcR was higher in the fourth-instar and fifth-instar larvae, a sudden decrease in sixth-instar larvae, the adult was at rock bottom. Both the testes and the ovaries, the expression of TeEcR gene was found to be gradually decreased from fourth instar to adult. The expression was significantly higher in ovaries than in testes during fourth-instar and fifth-instar larvae development stage.

## Discussion

In this study, a full-length cDNA sequence of unique EcR gene from the cricket *T.* *emma* was isolated and characterized. The cDNA sequence of TeEcR and its deduced amino acid sequence reflected a high degree of homology with the EcR homologs identified from other insects, indicating that this newly isolated cDNA encoded the cricket *T. emma* EcR protein. The TeEcR gene shared about 87–92% identity in amino acids sequence with the orthopteroidea insects *Blattella germanica* and *Locusta migratoria* EcRs. The EcRs of Coleoptra insects were all 80–89% range and 70–78% identity with Hymenoptera EcRs, which indicated the EcR gene follows conservative property in genetic and evolutionary pathways.

It is well known that amino acid sequences of the A/B region from EcRs are diverse that are produced by alternative promoting and splicing in arthropods (Lafont 2000b, Shirai et al. 2007). In addition, Talbot et al. (1993) reported that the fruitfly (*Drosophila melanogaster*) has three EcR isoforms: A, B1, and B2. The water flea (*D. magna*) also has three EcR isoforms (A1, A2, and B), and the isoforms have common regions including C- and E/F-region (Kato et al. 2007). Tan and Palli (2008) reported two EcR isoforms (A and B) in the red flour beetle (*T. castaneum*) and revealed that these EcR isoforms had distinct roles during metamorphosis. Although only one type of *TeEcR* was cloned using the RACE with the primers designed in C and E/F region, it was the same as other orthopteroidea insects (*Blattella germanica* and *Locusta migratoria*)*.* But the A/B domains of the *TeEcR* gene are fairly different from those of other arthropods, so it is difficult to be determined the exact *EcR* isoforms compared with the isoforms identified in other species.

The E regions of EcRs were considerably conserved in all species. The identity of TeEcR to those of Orthoptera was highest, followed by those to Coleoptera, as well as Hymenoptera (Fig. 2). The C-region sequences of EcRs were also highly conserved among several species, as shown in Table 2. In the C region, there are two zinc finger regions containing a P-box and a D-box, which are important for DNA recognition (Umesono and Evans 1989). The P-box of TeEcR is 100% identical with that of other insect EcRs. The D-box of TeEcR is 100% identical with that of orthopteran insects and is also highly homologous with that of Coleoptera (100% with *Tenebrio molitor*, 80% with *L. decemlineata*). However, it shows only 40% identity with the D-boxes of Lepidoptera and Diptera. Those may reveal their phylogenetic relationship.

Ecdysteroids play a pivotal role in development, growth, molt, and in the control of reproduction in the adult stage (Lafont 2000a, Tan and Palli 2008). EcR belongs to a molecular target of the ecdysteroids in arthropods, usually ecdysteroids play roles through EcR. The expression of the whole body and gonad at different development stage of TeEcR but different levels was detected in this experiment. TeEcR gene was found to be expressed at the higher level in the embryo, then significantly decreased in early larval (first to third instar), increased rapidly in late larval stage (fourth to sixth instar), and at last reduced in the adult. This gene expression pattern was similar to *Locusta migratoria* in development stage (David et al. 1998).

The expression of TeEcR in the female body is higher than male body during fourth- and fifth-instar larvae but is lower than male body in sixth-instar larvae and adult, which may be related with the cricket grouth, molt regulation, and morphological structure establishment. The expression of TeEcR pattern in ovaries was similar to the testes, but the expression was significantly higher in ovaries than testes in all stage particularly during fourth- and fifth-instar larvae. Those show that TeEcR may be very important for starting development of *T. emma* gonad, especially for the ovaries.

The expression patterns of *TeEcR* did not coincide with all peaks of ecdysteroid titer and were different depending on developing stages in other insects such as the fruitfly (Talbot et al. 1993), sheep blowfly (Hannan and Hill 1997), and red flour beetle (Tan and Palli 2008). These imply that the expression of *TeEcR* gene was not controlled by ecdysteroid only. The expression of another NR *TeERR* (estrogen relative receptor) had been studied in the crickets (He et al. 2010), and the expression pattern of *TeERR* was similar to *TeEcR*. Maybe there was some relationship between these two NR genes. But it certainly needs further study to confirm this hypothesis.

In summary, the results presented here on cDNAs encoding NR proteins from a primitive type insect will contribute to our understanding of the diversity of members of this ancient gene family and provide tools necessary for analysis of the regulation of NR gene expression. Our finding of differential expression of the *TeEcR* gene at distinct development stages of the body and gonad may indicate the physiological importance of *TeEcR* in the cricket. Also, the relationship between *TeEcR* and *TeERR* needs to be further indagated.
